# Micro-RNAs 518d-3p and 618 Are Upregulated in Individuals With Type 1 Diabetes With Multiple Microvascular Complications

**DOI:** 10.3389/fendo.2019.00385

**Published:** 2019-06-12

**Authors:** Daniele P. Santos-Bezerra, Aritania S. Santos, Gabriel C. Guimarães, Sharon N. Admoni, Ricardo V. Perez, Cleide G. Machado, Tatiana S. Pelaes, Marisa Passarelli, Ubiratan F. Machado, Marcia S. Queiroz, Maria Elizabeth R. da Silva, Maria Lucia Correa-Giannella

**Affiliations:** ^1^Laboratório de Carboidratos e Radioimunoensaio, LIM-18, Hospital das Clinicas HCFMUSP, Faculdade de Medicina da Universidade de São Paulo, São Paulo, Brazil; ^2^Divisão de Oftalmologia do Hospital das Clinicas, HCFMUSP, Faculdade de Medicina da Universidade de São Paulo, São Paulo, Brazil; ^3^Laboratório de Lipides, LIM-10, Hospital das Clinicas HCFMUSP, Faculdade de Medicina da Universidade de São Paulo, São Paulo, Brazil; ^4^Programa de Pós-Graduação em Medicina, Universidade Nove de Julho (UNINOVE), São Paulo, Brazil; ^5^Departamento de Fisiologia e Biofisica, Instituto de Ciencias Biomedicas, Universidade de São Paulo, São Paulo, Brazil; ^6^Divisão de Endocrinologia do Hospital das Clinicas, HCFMUSP, Faculdade de Medicina da Universidade de São Paulo, São Paulo, Brazil

**Keywords:** microvascular diabetes complications, epigenetics, micro-RNAs, diabetes kidney disease, diabetic retinopathy, peripheral neuropathy, cardiovascular autonomic neuropathy

## Abstract

**Objective:** To compare the serum micro-RNAs (miRNAs) profile of individuals with type 1 diabetes without microvascular complications vs. those with multiple severe microvascular complications, in order to identify epigenetically modulated pathways in these two groups of individuals.

**Research Design and Methods:** A total of 10 subjects were selected among individuals followed in the Diabetes Outpatient Clinic and sorted according to the absence or presence of all microvascular complications. Samples from these participants were used for evaluation of serum miRNA expression profile employing a qRT-PCR assay with hydrolysis probes based on the Taqman Low Density Arrays (TLDA) system. The top six most differentially expressed miRNAs between the aforementioned groups were validated by qRT-PCR in additional 47 type 1 diabetes individuals sorted according to the absence or presence of all microvascular complications and matched for age, sex, degree of metabolic control, diabetes duration, and age at diagnosis.

**Results:** Twenty one out of three hundred and seventy seven miRNAs were upregulated in the group of individuals with all microvascular complications vs. the group without complications. The following miRs were validated: 518-3p, 34a-5p, 126-5p, 425-5p, 618, and 139-5p and logistic regression analyses showed that miRNA-518-3p and miRNA-618 were positively associated with multiple microvascular complications after adjustment for age, sex, diabetes duration, HbA_1_c and use of statin, angiotensin-converting enzyme inhibitors and amlodipine.

**Conclusions:** In this cohort of type 1 diabetes individuals, serum miR-518d-3p and miR-618 were upregulated in those with diabetes kidney disease, diabetes retinopathy, peripheral neuropathy, and cardiovascular autonomic neuropathy in comparison to individuals with no microvascular complications.

## Introduction

Diabetes kidney disease (DKD), diabetic retinopathy (DR), peripheral neuropathy (PN), and cardiovascular autonomic neuropathy (CAN) have hyperglycemia as the main etiopathogenic factor for their development (1). Several studies have already shown that periods of hyperglycemia result in permanent abnormalities in the target tissues of complications, a phenomenon known as “metabolic memory” (2, 3). Post-translational modifications of histones, cytosine methylation, and the action of micro-RNAs (miRNAs) explain deleterious effects of hyperglycemia even after improved glycemic control (4).

miRNAs constitute a class of small RNA with about 19 to 25 non-coding nucleotides that is the main incorporated component of the RNA-induced silencing complex (RISC), which represses translation or degrades target mRNAs (5). Besides acting as post-transcriptional regulators of gene expression, miRNAs can indirectly modulate DNA methylation and even stimulate transcription after binding to promoter regions of target-genes (5).

miRNAs are present in the bloodstream, possibly originated from passive leakage of damaged/dead cells or from active cell secretion, either in exosomes or linked to proteins (6). Secreted miRNAs may play a role in intercellular communication, so that donor cells can affect gene expression of distant or adjacent target-cells. Thus, beyond acting as hormones, serum miRNAs can be functional biomarkers for pathological conditions (7). Several studies have shown that a significant portion of the circulating miRNAs is not in microvesicles but it is associated with the Argonauta 2 (AGO2) protein, the main protein of the RISC complex, and this association explains the stability of circulating miRNAs (8, 9).

Most studies with miRNAs in the context of diabetes have been performed in animal models or in cell culture, but there are already studies addressing the association of serum miRNAs with one or more microvascular complication (10–14). However, there are no studies comparing serum miRNAs between type 1 diabetes individuals without vs. with all microvascular complications. Thus, in the present study we compared the serum miRNA profile of individuals with type 1 diabetes without microvascular complications vs. those with multiple severe microvascular complications: DKD, DR, PN, and CAN, in order to identify epigenetically modulated pathways in these two groups of individuals.

## Materials and Methods

### Participants

A total of 10 individuals were selected among type 1 diabetes individuals followed in the Diabetes Outpatient Clinic of Hospital das Clinicas da Faculdade de Medicina da Universidade de Sao Paulo and sorted according to the absence or presence of microvascular complications: (1) *n* = 5 individuals without DKD (estimated glomerular filtration rate [eGFR] > 90 mL/min/1.73 m^2^ and urinary albumin excretion <30 mg/g creatinine), without DR, without PN, and without CAN; and (2) *n* = 5 individuals with DKD (eGFR < 60 mL/min /1.73 m^2^ and urinary albumin excretion ≥300 mg/g creatinine), with moderate or severe DR, with PN, and with CAN. The two groups were matched as far as possible for age, sex, and degree of metabolic control (Table 1). Samples from these participants were used for the evaluation of serum miRNA expression profile.

**Table 1:** Characteristics of type 1 diabetes individuals evaluated in the micro-RNA expression profile.

	**Without microvascular complications**	**With multiple microvascular complications**
**Clinical and biochemical characteristics**		
*N*	5	5
Age (years)	37 (28–44)	30 (26–34)
Sex (% female)	60	60
BMI (kg/m^2^)	21.6 (19.9–24.7)	23.4 (22.9–23.9)
Arterial Hypertension (%)	0	40
Total cholesterol (mg.dL^−1^)	170 (167–181)	158 (137–194)
HDL cholesterol (mg.dL^−1^)	75 (65–86)	56 (49–65)
LDL cholesterol (mg.dL^−1^)	88 (72–94)	85 (67–114)
Triglycerides (mg.dL^−1^)	55 (51–96)	64 (58–99)
eGFR	100 (78–114)	19 (14–49)
**Diabetes status**		
Diabetes duration (years)	20 (15–31)	23 (14–24)
Age at diagnosis (years)	12 (6–25)	8 (7–15)
HbA_1_C (%)	8.5 (7.7–8.7)	8.6 (7–11.7)

*Results expressed as median and interquartile range and % of cases (categorical variables). HbA1c is expressed in % of total hemoglobin. BMI, body mass index; eGFR, estimated glomerular filtration rate*.

The top six most differentially expressed miRNAs between the aforementioned groups were further validated in additional 47 type 1 diabetes individuals sorted according to the absence or presence of microvascular complications, using the same criteria described above: (1) *n* = 20 individuals without DKD, DR, PN and, CAN; and (2) *n* = 27 individuals with DKD, moderate or severe DR, PN, and CAN. The two groups were matched for age, sex, degree of metabolic control, diabetes duration, and age at diagnosis. The lower body mass index (BMI) and the higher frequency of arterial hypertension in the group with multiple microvascular complications reflect the presence of advanced DKD (Table 2).

**Table 2:** Characteristics of type 1 diabetes individuals evaluated in the validation of differentially expressed micro-RNAs.

	**Without microvascular complications**	**With multiple microvascular complications**	***P*-value**
**Clinical and biochemical characteristics**			
*N*	20	27	
Age (years)	33 (26–46)	34 (30–40)	NS
Sex (% female)	70	67	NS
BMI (kg/m^2^)	23.4 (22.0–25.8)	21.3 (19.2–23.5)	0.01
Arterial Hypertension (%)	20	50	0.04
Total cholesterol (mg.dL^−1^)	166 (153–189)	163 (142–188)	NS
HDL cholesterol (mg.dL^−1^)	72 (64–84)	56 (40–72)	NS
LDL cholesterol (mg.dL^−1^)	87 (72–103)	88 (73–107)	NS
Triglycerides (mg.dL^−1^)	65 (51–82)	82 (55–114)	NS
eGFR	107 (91–126)	26 (10–52)	0.001
**Diabetes status**			
Diabetes duration (years)	17 (13–28)	22 (16–29)	NS
Age at diagnosis (years)	14 (11–18)	12 (8–16)	NS
HbA_1_C (%)	8.15 (7.3–9.2)	8.26 (7–9.8)	NS

*Results expressed as median and interquartile range and % of cases (categorical variables). HbA1c is expressed in % of total hemoglobin. BMI, body mass index; eGFR, estimated glomerular filtration rate; NS, not significant*.

The study was conducted in compliance with the Declaration of Helsinki, in accordance with the Institutional Ethics Committees. After signing informed consent, the participants were evaluated for clinical and biochemical characteristics and for the status of DKD, DR, PN, and CAN as previously described (10).

### Serum miRNA Expression Profile

Serum miRNA profile was performed in four steps. Firstly, peripheral blood from the 10 type 1 diabetes individuals was collected in a yellow cap BD tube (Becton Dickinson, Franklin Lakes, USA) specific for serum separation. Tubes were centrifuged at 4°C for 20 min at 1,500 g and serum was separated, aliquoted, and frozen at −80°C. All 10 individuals' sera were analyzed in NanoDrop™ 2000 Spectrophotometer (NanoDrop, Rockland, USA) for the degree of hemolysis at 414 nm (absorbance peak of free hemoglobin) and all presented reading <0.2, being ready for the next step (15, 16), the extraction of total miRNA by the *miRNeasy Mini commercial kit* (Qiagen, Hilden, Germany).

Before the extraction, 3.5 μL of *miRNeasy Serum/Plasma SpikeIn Control* (Qiagen) were added to 200 μL of each serum sample, corresponding to 1.6 ×10^8^ copies/μL of miR-39, which was used as an exogenous positive control.

The amount and quality of the extracted miRNAs were evaluated by spectrophotometer ND-1000. A quantitative real-time polymerase chain reaction (qRT-PCR) after reverse transcription was performed for miR-39 using *Taqman*® *miR-39 assay* (ThermoFisher Scientific, Carlsbad, USA) and *Taqman*® *Master Mix Universal II at UNG* (ThermoFisher Scientific) and confirmed the maintenance and integrity of the extracted miRNAs.

For synthesis and pre-amplification of complementary DNA (cDNA) from total miRNA, the *Megaplex*™ *RT Primers, Human Pool A v2.1* (Thermo Fisher Scientific) and the *Thermal cycler Veriti 96 WellsCycler* (Thermo Fisher Scientific) were used according to the manufacturer's instructions.

T*aqman*® *Human MicroRNA Array A kit* (ThermoFisher Scientific) and *Taqman*® *Universal Master Mix II at UNG* (ThermoFisher Scientific) were used for evaluation of serum miRNAs profile. The *Taqman*® *Human MicroRNA Array A kit* is a qRT-PCR assay with hydrolysis probes based on the Taqman Low Density Arrays (TLDA) system containing Taqman probes sufficient for expression analysis of 377 miRNAs, in addition to four endogenous controls, totaling 381 miRNAs. The experiment was performed with Viia 7 (Thermo Fisher Scientific).

### Validation of Differentially Expressed miRNAs

The top six most differentially expressed miRNAs in the serum profile were validated in serum samples from 47 type 1 diabetes individuals by qRT-PCR. Extraction and evaluation of the miRNAs were performed as described above. Synthesis and pre-amplification of cDNA was performed with *TaqMan*™ *Advanced miRNA cDNA Synthesis kit* (Thermo Fisher Scientific) and *Thermocycler Veriti 96-Well Thermal Cycler* (Thermo Fisher Scientific) according to the manufacturer's instructions.

*Taqman*® *Fast Advanced Master Mix* (Thermo Fisher Scientific) and *TaqMan*® *Advanced miRNA Assays* (Thermo Fisher Scientific) were used for real-time PCR reaction in StepOne Plus (Thermo Fisher Scientific).

miRNAs content was calculated as relative expression units (2^−Δ*Ct*^) normalized by the exogenous cel-miR-39 spike-in control and log10 transformed for statistical analysis.

### Statistical Analysis

**Figure 1:**
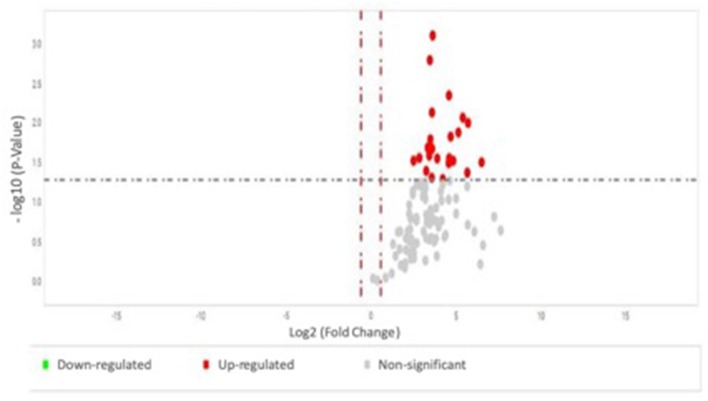
Volcano plot on the results of two-way ANOVA test comparing serum miRNA expression profile between individuals without vs. with complications, with the X axis representing the fold change and the Y axis representing the *P*-value. The group without complications was used as reference; each dot represents one micro-RNA.

Results are expressed as median and interquartile range. Clinical and biochemical differences between groups were assessed by Pearson's chi-squared test and Wilcoxon/Kruskal-Wallis test. A volcano plot on the results of two-way ANOVA test comparing serum miRNA expression profile between individuals without vs. with complications was generated, with the X axis representing the fold change and the Y axis representing the ANOVA *P*-value. Cutoff lines were added at−1.5 to 1.5 on the X axis and 0.05 on the Y axis (Figure 1). For the six differentially expressed miRNAs validated, likelihood ratio tests were employed to test models with and without the following covariates: sex, age, diabetes duration, HbA1c, and medicines whose frequency of use was significantly different between the groups with and without complications: statins, angiotensin-converting enzyme inhibitors (ACEI) and amlodipine; a *P* < 0.05 was considered significant. Statistics were performed with the JMP software (SAS Institute Inc., Cary, USA).

## Results

### Serum miRNA Expression Profile

Of the 377 target miRNAs evaluated, 193 miRNAs were expressed in the serum of individuals with type 1 diabetes. A total of 21 miRNAs presented a significantly higher expression in the group of individuals with all microvascular complications vs. the group without complications. Table 3 shows these 21 miRNAs ranked in order of decreasing significance.

**Table 3:** Differentially expressed serum micro-RNAs between type 1 diabetes individuals without vs. with multiple microvascular complications.

**Micro-RNA**	**Fold change**	***P***
hsa-miR-518d-3p	3.640274	0.001
hsa-miR-34a-5p	3.472748	0.002
hsa-miR-126-5p	4.599853	0.004
hsa-miR-425-5p	3.600389	0.007
hsa-miR-618	5.414643	0.008
hsa-miR-139-5p	5.724132	0.010
hsa-miR-181b-5p	5.152832	0.013
hsa-let-7d-5p	4.698996	0.014
hsa-miR-92a-3p	3.501949	0.015
hsa-miR-30c-5p	3.605731	0.020
hsa-miR-574-3p	3.35177	0.020
hsa-miR-150-5p	3.445991	0.022
hsa-miR-106a-5p	3.441085	0.025
hsa-miR-346	2.854594	0.026
hsa-miR-548a-3p	4.612883	0.026
hsa-miR-26a-5p	3.907852	0.027
hsa-miR-28-3p	4.849249	0.029
hsa-miR-342-3p	2.513238	0.029
hsa-miR-130a-3p	6.521318	0.030
hsa-let-7e-5p	4.588805	0.039
hsa-miR-483-5p	3.260327	0.049

*hsa, Homo sapiens; P ≤ 0.05 was considered statistically significant. Fold change is calculated in log_2_*.

### Validation of Differentially Expressed miRNAs

Out of the 21 differentially expressed miRNAs, the following miRs were validated by qRT-PCR: 518-3p, 34a-5p, 126-5p, 425-5p, 618, and 139-5p. Logistic regression analyses showed that miRNA-518-3p and miRNA-618 were positively associated with multiple microvascular complications in the unadjusted model (Model 1), after adjustment for age and sex (Model 2), for age, sex, diabetes duration, and HbA_1_c (Model 3) and for sex, age, diabetes duration, HbA1c and use of statin, ACEI and amlodipine (Model 4) (Table 4).

**Table 4:** Odds ratios for multiple microvascular complications by the six validated micro-RNAs.

		**miRNA 518d**	**miRNA 34a**	**miRNA 126**	**miRNA 425**	**miRNA 618**	**miRNA139**
Model 1	Unadjusted	OR = 3.75 (1.37–12.67) *P* = 0.0088	OR = 2.38 (0.59–11.01) *P =* 0.2266	OR = 1.47 (1.02–2.69) *P =* 0.2319	OR = 1.87 (0.67–5.64) *P =* 0.8733	OR = 4.11 (1.32–15.41) *P =* 0.0131	OR = 1.87 (0.67–5.64) *P =* 0.1785
Model 2	Adjusted for sex and age	OR = 4.28 (1.51–14.94) *P =* 0.0049	OR = 2.74 (0.66–13.06) *P =* 0.1682	OR = 1.93 (0.68–5.96) *P =* 0.2153	OR = 1.03 (0.42–2.56) *P =* 0.9463	OR = 4.45 (1.39–17.54) *P =* 0.0102	OR = 2.64 (0.60–12.98) *P =* 0.2001
Model 3	Adjusted for sex, age, diabetes duration, and HbA_1_c	OR = 3.43 (1.16–12.38) *P =* 0.0248	OR = 1.58 (0.31–8.36) *P =* 0.5766	OR = 1.80 (0.59–6.00) *P =* 0.3084	OR = 0.97 (0.37–2.55) *P =* 0.9493	OR = 3.97 (1.19–16.94) *P =* 0.0237	OR = 2.10 (0.47–10.33) *P =* 0.3348
Model 4	Adjusted for sex, age, diabetes duration, HbA_1_c, and use of statin, ACEI, and amlodipine	OR = 10.6 (2.05–141.1) *P =* 0.0027	OR = 2.38 (0.33–19.1) *P =* 0.3882	OR = 2.82 (0.66–14.43) *P =* 0.1598	OR = 0.74 (0.20–2.63) *P =* 0.6387	OR = 17.80 (2.61–258.15) *P =* 0.0017	OR = 2.43 (0.36–19.48) *P =* 0.3596

*ACEi, angiotensin-converting enzyme inhibitors; miRNA, microRNA; OR, odds ratio*.

*In silico* analyses in the miRDB dataset did not reveal common target-genes between these two miRNAs; there are 31 target-genes for miR-518d-3p and 225 target-genes for miR-618.

## Discussion

The present study identified two miRNAs upregulated in type 1 diabetes individuals with all chronic microvascular complications in relation to individuals with no microvascular complications, miR-518d-3p and miR-618, widening the spectrum of epigenetic changes in the setting of diabetes complications.

miR-518 was identified as a miRNA potentially targeting peroxisome proliferator-activated receptor alpha (PPARα) (17). A functional assay confirmed that miR-518d directly targets PPARα by binding to the 3'-UTR of its mRNA in a study that also suggested that increased miR-518d expression in placenta is implicated in gestational diabetes (18).

PPARα is a nuclear receptor that plays a critical role in lipid homeostasis and inflammation (18). It has been shown that PPARα is decreased in the retina of experimental models of diabetes and that the PPARα knockout worsened DR (19). On the other hand, PPARα has anti-apoptotic and antioxidant effects in the ischemic retina (20), and there are studies showing a lower incidence of DR in individuals exposed to fibrates, which are PPARα agonists (21–23).

Thus, we hypothesized that miR-518d-3p is involved in the PPARα downregulation reported in DR and that the upregulation of miR-518d-3p observed in the group presenting all microvascular complications may reflect moderate to severe DR of individuals included in this group. An additional finding that suggests the involvement of miR-518d-3p in DR is its increased expression in human retinal pigment cells exposed to oxidative stress (24). It is interesting to mention that another miRNA, miR-21, was found to be overexpressed in the retina of a type 2 diabetes rodent model and at least partially responsible for PPARα downregulation (25).

It is also possible that upregulation of miR-518d-3p be a marker of generalized endothelial dysfunction in these individuals since the expression of this miRNA increased in human endothelial cells exposed to acrolein, an unsaturated aldehyde able to induce oxidative stress and inflammation (26), conditions also triggered by hyperglycemia (27).

Regarding miRNA-618, its deregulation has been associated with several neoplasias (28). It has also been identified as a miRNA induced by hypoxia in human primary pulmonary artery smooth muscle cells (29) and upregulated in the blood of individuals during the acute phase of Kawasaki disease, a vasculitis in which transforming growth-factor β (TGFβ) is implicated (30). Among the predicted targets for miR-618 are the following seven genes belonging to TGFβ pathway, *TGFB2, THBS1, NOG, KLF10, PPP2CA, PPP2CB*, and *ID2*, according to miRDB database. Given the involvement of TGFβ in diabetes complications (31), further studies are necessary to elucidate whether miR-618 upregulation is related to deregulation of the TGFβ pathway in the setting of diabetes complications.

None of the 21 miRNAs upregulated in the group with all microvascular complications was reported to be associated with hyperglycemia in a previous study of circulating miRNAs in individuals with type 1 diabetes. In addition, the expression of the six validated microRNAs did not differ significantly when individuals were sorted by HbA1c tertiles (data not shown).

Three of the 21 miRNAs upregulated in the group with all microvascular complications (miR-139-5p, miR-106a, and miR-574-3p) were previously identified as upregulated in type 1 diabetes individuals with one or more complications (cardiovascular diseases and/or DR and/or albuminuria) in comparison to individuals with no complications (11), while four miRNAs upregulated in the present study were found downregulated in that study (miR-92a, miR-126, miR-483-5p, and miR-150) (11). These findings suggest that the serum miRNA profile may be sensitive to the presence or absence of specific chronic complications.

This study must be interpreted in light of limitations of cross-sectional studies and requires confirmation in larger cohorts. Among the study's strengths are the use of individual samples (as opposed to pooled serum samples) in the screening phase, the systematic evaluation of all microvascular complications (including CAN) and the homogeneity of the two evaluated groups.

In conclusion, in this cohort of type 1 diabetes individuals, serum miR-518d-3p and miR-618 are upregulated in those with diabetes kidney disease, diabetes retinopathy, peripheral neuropathy and cardiovascular autonomic neuropathy in comparison to individuals with no microvascular complications.

## Ethics Statement

The study was conducted in compliance with the Declaration of Helsinki, in accordance with the Comitê de Ética e Pesquisa (CAPPesq) do Hospital das Clínicas da Faculdade de Medicina da Universidade de São Paulo (#1.433.880). All participants provided written informed consent prior to sample collection.

## Author Contributions

DS-B collected and analyzed clinical and biochemical data, performed miRNA experiments, wrote, reviewed, and edited the manuscript. AS performed miRNA experiments and reviewed the manuscript. GG collected clinical and biochemical data, performed miRNA extraction, and reviewed the manuscript. SA performed neuropathy evaluation and reviewed the manuscript. RP and CM performed retinopathy evaluation and reviewed the manuscript. TP collected clinical and biochemical data and reviewed the manuscript. MP, UM, and MdS participated in study design and reviewed the manuscript. MQ collected clinical data and reviewed the manuscript. MC-G designed the study, wrote, reviewed and edited the manuscript.

### Conflict of Interest Statement

The authors declare that the research was conducted in the absence of any commercial or financial relationships that could be construed as a potential conflict of interest.
